# 2510: QIPR: Creating a Quality Improvement Project Registry

**DOI:** 10.1017/cts.2017.85

**Published:** 2018-05-10

**Authors:** Amber L. Allen, Christopher Barnes, Kevin S. Hanson, David Nelson, Randy Harmatz, Eric Rosenberg, Linda Allen, Lilliana Bell, Lynne Meyer, Debbie Lynn, Jeanette Green, Peter Iafrate, Matthew McConnell, Patrick White, Samantha Davuluri, Tarun Gupta Akirala

**Affiliations:** 1 University of Florida Clinical and Translational Science Institute, Gainesville, FL, USA;; 2 UF Health Sebastian Ferrer, Gainesville, FL, USA

## Abstract

OBJECTIVES/SPECIFIC AIMS: To create a searchable public registry of all Quality Improvement (QI) projects. To incentivize the medical professionals at UF Health to initiate quality improvement projects by reducing startup burden and providing a path to publishing results. To reduce the review effort performed by the internal review board on projects that are quality improvement Versus research. To foster publication of completed quality improvement projects. To assist the UF Health Sebastian Ferrero Office of Clinical Quality & Patient Safety in managing quality improvement across the hospital system. METHODS/STUDY POPULATION: This project used a variant of the spiral software development model and principles from the ADDIE instructional design process for the creation of a registry that is web based. To understand the current registration process and management of quality projects in the UF Health system a needs assessment was performed with the UF Health Sebastian Ferrero Office of Clinical Quality & Patient Safety to gather project requirements. Biweekly meetings were held between the Quality Improvement office and the Clinical and Translational Science – Informatics and Technology teams during the entire project. Our primary goal was to collect just enough information to answer the basic questions of who is doing which QI project, what department are they from, what are the most basic details about the type of project and who is involved. We also wanted to create incentive in the user group to try to find an existing project to join or to commit the details of their proposed new project to a data registry for others to find to reduce the amount of duplicate QI projects. We created a series of design templates for further customization and feature discovery. We then proceed with the development of the registry using a Python web development framework called Django, which is a technology that powers Pinterest and the Washington Post Web sites. The application is broken down into 2 main components (i) data input, where information is collected from clinical staff, Nurses, Pharmacists, Residents, and Doctors on what quality improvement projects they intend to complete and (ii) project registry, where completed or “registered” projects can be viewed and searched publicly. The registry consists of a quality investigator profile that lists contact information, expertise, and areas of interest. A dashboard allows for the creation and review of quality improvement projects. A search function enables certain quality project details to be publicly accessible to encourage collaboration. We developed the Registry Matching Algorithm which is based on the Jaccard similarity coefficient that uses quality project features to find similar quality projects. The algorithm allows for quality investigators to find existing or previous quality improvement projects to encourage collaboration and to reduce repeat projects. We also developed the QIPR Approver Algorithm that guides the investigator through a series of questions that allows an appropriate quality project to get approved to start without the need for human intervention. RESULTS/ANTICIPATED RESULTS: A product of this project is an open source software package that is freely available on GitHub for distribution to other health systems under the Apache 2.0 open source license. Adoption of the Quality Improvement Project Registry and promotion of it to the intended audience are important factors for the success of this registry. Thanks goes to the UW-Madison and their QI/Program Evaluation Self-Certification Tool (https://uwmadison.co1.qualtrics.com/SE/?SID=SV_3lVeNuKe8FhKc73) used as example and inspiration for this project. DISCUSSION/SIGNIFICANCE OF IMPACT: This registry was created to help understand the impact of improved management of quality projects in a hospital system. The ultimate result will be to reduce time to approve quality improvement projects, increase collaboration across the UF Health Hospital system, reduce redundancy of quality improvement projects and translate more projects into publications.

